# Articulating Materials Are Determinants of Survivorship of Hip Arthroplasties Performed for Nontraumatic Osteonecrosis of the Femoral Head

**DOI:** 10.3390/ma18092125

**Published:** 2025-05-06

**Authors:** Seneki Kobayashi, Nobuhiko Sugano, Wataru Ando, Wakaba Fukushima, Kyoko Kondo, Takashi Sakai

**Affiliations:** 1 The Investigation Committee on Osteonecrosis of the Femoral Head Under the Ministry of Health, Labour and Welfare, Tokyo 100-8959, Japan; sugano@ort.med.osaka-u.ac.jp (N.S.); w-ando@umin.ac.jp (W.A.); wakaba@omu.ac.jp (W.F.); cozy@yamaguchi-u.ac.jp (T.S.); 2 Department of Orthopaedic Surgery, Suwa Red Cross Hospital, Suwa 392-8510, Japan; 3 Department of Orthopaedic Medical Engineering, Osaka University Graduate School of Medicine, Suita 565-0871, Japan; 4 Department of Orthopaedic Surgery, Kansai Rosai Hospital, Amagasaki 660-0064, Japan; 5 Department of Public Health, Osaka Metropolitan University Graduate School of Medicine, Osaka 545-8585, Japan; 6 Research Support Platform, Osaka Metropolitan University Graduate School of Medicine, Osaka 545-8585, Japan; kyou@omu.ac.jp; 7 Department of Orthopedic Surgery, Yamaguchi University Graduate School of Medicine, Ube 755-8505, Japan

**Keywords:** osteonecrosis of the femoral head (ONFH), total hip arthroplasty (THA), bipolar hemiarthroplasty, alumina bipolar hemiarthroplasty, metal bipolar hemiarthroplasty, surgical approach, minimum-incision surgery (MIS), survival rate

## Abstract

A nationwide multicenter follow-up cohort study of hip-replacement arthroplasties performed for nontraumatic osteonecrosis of the femoral head (ONFH) was conducted to clarify factors associated with need for reoperation. We analyzed 7393 arthroplasties including 6284 total hip arthroplasties (THAs), 886 bipolar hemiarthroplasties (BPs), 188 total resurfacing arthroplasties, and 35 hemi-resurfacing arthroplasties (hRSs). The identified risk factors were combined systemic steroid use and excessive alcohol consumption (both ONFH-associated factors), a lateral approach, alumina BPs (aBPs), and hRSs, which were reported previously. The present study performed further analyses separately for THAs and BPs to clarify risk factors in each surgical group. A Cox proportional-hazard model identified the following risk factors: the acetabular-articulating materials of conventional polyethylene (cPE) and metal in the THAs and both ONFH-associated factors, minimum-incision surgery (MIS), and aBPs in the BPs. The risk factors were specific to each surgical group. In the ONFH patients, when performing THA, cPE and metal are not recommended as the acetabular-articulating material. When performing BP, patients with both ONFH-associated factors should be treated carefully, and the employment of MIS and use of aBP are not good strategies.

## 1. Introduction

Nontraumatic osteonecrosis of the femoral head (ONFH) patients undergoing hip-replacement arthroplasties are generally younger, more often of the male gender [1], and at higher risks of postoperative dislocation [2,3] and a need for reoperation [4,5,6,7,8], compared with patients undergoing the procedures mainly for osteoarthrosis (OA). Hip arthroplasty practice has changed noticeably. In total hip arthroplasty (THA), acetabular liners made of highly (approximately 10 Mrad) cross-linked polyethylene (HXLPE) and larger prosthetic heads have increasingly been used. In bipolar hemiarthroplasties (BPs), usually performed for ONFH before the development of OA, many modifications have been made to improve their poor results [9,10]. To reduce osteolysis due to polyethylene debris generated by neck–outer head impingement in BP [11,12], a new type of BPs (nBPs) with a smooth, small-diameter (approximately 10 mm) neck without any sharp corners began to replace other BPs (oBPs) [1]. As contemporary femoral prostheses tended to have a neck with a larger diameter, ‘small-diameter’ was excluded from the definition, which described BPs with a smooth neck (sBPs) and the others with a rough-surfaced neck (rBPs). Alumina BPs (aBPs, with the outer surface of the outer head made of alumina ceramic) were developed to surpass the durability of metal BPs (mBPs). BPs with HXLPE in the outer head (hBPs) were also developed to improve the survivorship of BPs with conventional polyethylene (cPE) (cBPs). However, their efficacy has not been clinically proven. Therefore, hip-replacement arthroplasties performed for OFNH should be monitored; this was conducted in a nationwide multicenter follow-up cohort study.

In a survey conducted in 2023, we analyzed arthroplasties including THAs, BPs, total resurfacing arthroplasties (tRSs), and hemi-resurfacing arthroplasties (hRSs). The identified risk factors were combined systemic steroid use and excessive alcohol consumption (both ONFH-associated factors), a lateral approach, aBPs, and hRSs, which were reported previously [13]. However, it was not clear which risk factors were applicable to which surgical treatments. Our hypothesis was that risk factors could be specific to each surgical treatment. Therefore, the present study performed further analyses separately for THAs and BPs to clarify risk factors in each surgical group.

## 2. Materials and Methods

### 2.1. THAs and BPs Analyzed for Factors Related to Need for Reoperation

**Figure 1:**
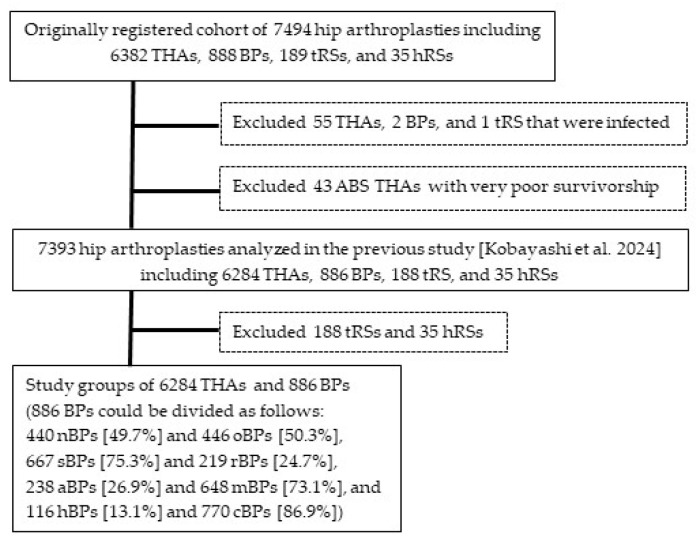
The flowchart of selecting the study groups of 6284 primary total hip arthroplasties (THAs) and 886 bipolar hemiarthroplasties (BPs) from the originally registered cohort of 7494 hip arthroplasties. tRSs: total resurfacing arthroplasties; hRSs: hemi-resurfacing arthroplasties; nBPs: a new type of BPs; oBPs: other BPs; sBPs: BPs with a smooth neck; rBPs: other BPs with a rough-surfaced neck; mBPs: BPs with a metal outer head; and aBPs: BPs with an alumina outer head. Kobayashi et al. 2024 [13].

The previous study [13] described the method of the nationwide multicenter follow-up cohort study of hip-replacement arthroplasties performed for ONFH and the demographic and operative details of the 7393 arthroplasties (after exclusion of 58 infected hips and 43 ABS THAs with very poor survivorship from the entire cohort of 7494 arthroplasties observed in the 2023 survey) that were analyzed for factors related to need for reoperation. They included 6284 THAs (85.0%), 886 BPs (12.0%), 188 tRSs (2.5%), and 35 hRSs (0.5%) (Figure 1). THAs and tRSs were performed in all ONFH stages, while BPs and hRSs were performed mainly before stage 4 (OA). The 886 BPs could be divided into two in four ways. As for hip prostheses and their providers, 81 types of acetabular components were provided by 15 companies and 137 types of femoral components by 18 companies.

### 2.2. Statistical Analyses

Factors related to need for reoperation were analyzed with a Cox proportional-hazard model using IBM SPSS statistics version 29 (IBM Corp, Armonk, NY, USA). Univariate analyses were first performed by applying the model to each of the demographic and operative variables with a significance level of *p* < 0.1. Variables with *p* < 0.1 were then examined together using the model with a significance level of *p* < 0.05 (multivariate analysis). Effects of the identified risk factors on the survivorship of hip arthroplasties were illustrated with the Kaplan–Meier estimator (with log-rank tests) with a significance level of *p* < 0.05. A χ^2^ test was used to compare the prevalence of categorical data and a *t*-test was used to compare means with a significance level of *p* < 0.05.

### 2.3. Ethical Approvals

Ethical approvals for this study were comprehensively obtained at three representative institutions: Shinshu University School of Medicine (8 January 2008, No. 1043), Suwa Red Cross Hospital (27 November 2014, No. 26–23 and 26 March 2019, No. 30–19), and Osaka University Graduate School of Medicine (29 January 2021, No. 20461). This study was carried out in accordance with the World Medical Association Declaration of Helsinki. All participants included in the study gave informed consent and agreed to participate in this study and to have their data published in a journal. 

## 3. Results

### 3.1. Comparison of Demographic and Surgical Variables Between THAs and BPs

The demographic and operative features of the 6284 THAs and the 886 BPs and their comparisons are listed in Table 1. They were different between the two surgical groups except for patient weight.

### 3.2. Analyses of the 6284 THAs

#### 3.2.1. Cox Proportional-Hazard Model Analyses

The follow-up of the 6284 THAs ranged from 0.1 to 27 (mean of 6.5) years, during which 189 hips (3.0%) needed reoperation. The univariate analyses identified five of the variables listed in Table 1 with *p* < 0.1 (Table 2). However, in the χ^2^ tests, incision length, femoral head material, and head diameter were strongly related (*p* < 0.001). THAs with a 22 mm prosthetic head were performed with conventional incision in 92.2% of cases, and the 22 mm heads were made of metal in 78.2% of cases. Therefore, incision length and head material were not included in the next multivariate analyses. As the remaining three variables had been reported concerning the survivorship of THAs and without a strong relationship between them, they were examined together with the model.

The multivariate survivorship analysis identified only acetabular-articulating material as a risk factor with *p* < 0.05 (Table 3). Compared with HXLPE or MXLPE, cPE and metal (cobalt–chrome or metal-on-metal THA) had inferior survivorship (*p* < 0.001 and *p* = 0.028, respectively), whereas that of ceramic (ceramic-on-ceramic THA) did not differ (*p* = 0.765).

#### 3.2.2. Survivorship Illustrated with Kaplan–Meier Estimator

**Figure 2:**
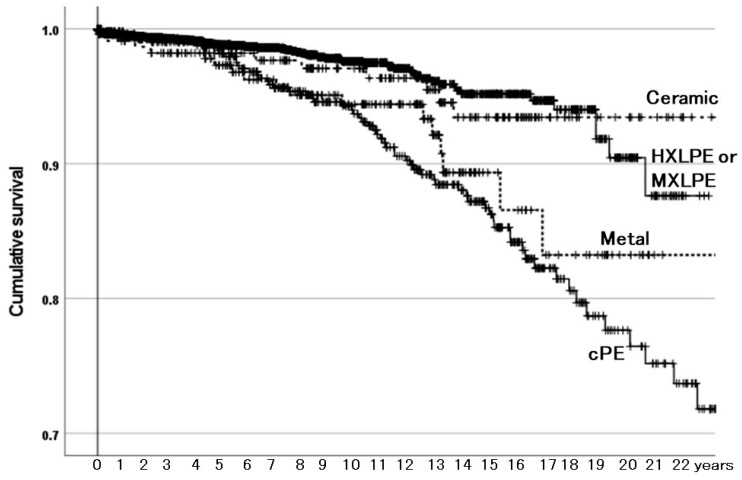
The cumulative survival of total hip arthroplasties (THAs) among types of acetabular-articulating materials with need for reoperation as the endpoint. HXLPE: highly (approximately 10 Mrad) cross-linked polyethylene. MXLPE: moderately (5 to 7.5 Mrad) cross-linked polyethylene. cPE: conventional polyethylene. Survival rates for ceramic, HXLPE or MXLPH, metal, and cPE were 97%, 98%, 94%, and 93% at 10 years and 93%, 90%, 83%, and 78% at 20 years, respectively. Compared with the HXLPE or MXLPH group, the metal and cPE groups had inferior survivorship (*p* = 0.004 and *p* < 0.001, respectively), but the ceramic group did not (*p* = 0.657).

The Kaplan–Meier estimator illustrated the effects of the acetabular-articulating materials on the survivorship of THAs with need for reoperation as the endpoint (Figure 2). Compared with the HXLPE or MXLPH group, the cPE and metal (metal-on-metal THA) groups had inferior survivorship, but the ceramic (ceramic-on-ceramic THA) group did not. The rates of need for reoperation were also different among them in a χ^2^-test (*p* < 0.001): 1.8% in the HXLPE or MXLPE group, 11.6% in the cPE group, 6.6% in the metal group, and 4.3% in the ceramic group.

Regarding reasons for need for reoperation, in the HXLPE or MXLPE group, recurrent dislocation was at the top, followed by periprosthetic femoral fracture (Table 4). In the cPE group, the most frequent three reasons were osteolysis, polyethylene wear and/or breakage, and recurrent dislocation. The top reason was adverse reaction to metal debris (ARMD) in the metal (metal-on-metal THA) group and recurrent dislocation in the ceramic (ceramic-on-ceramic THA) group.

### 3.3. Analyses of the 886 BPs

#### 3.3.1. Cox Proportional-Hazard Model Analyses

The follow-up of the 886 BPs ranged from 0.1 to 27 (mean, 9.6) years, during which 47 hips (5.3%) needed reoperation. The univariate analyses identified three of the variables listed in Table 1 with *p* < 0.1, i.e., ONFH-associated factors, incision length, and aBPs (Table 5). Without strong relationship among them, they were examined together with the model.

In the multivariate survivorship analysis, all the three variables were confirmed as risk factors with *p* < 0.05 (Table 6). Combined systemic steroid use and excessive alcohol consumption (both ONFH-associated factors) had a higher risk, with no associated factors as a reference (*p* = 0.008). Compared with conventional incision, minimal-incision surgery (MIS, defined as the use of a ≤ 10 cm incision to complete a hip arthroplasty) had a higher risk (*p* < 0.001). The aBPs had a higher risk than the mBPs (*p* = 0.004).

#### 3.3.2. Survivorship Illustrated with Kaplan–Meier Estimator

**Figure 3:**
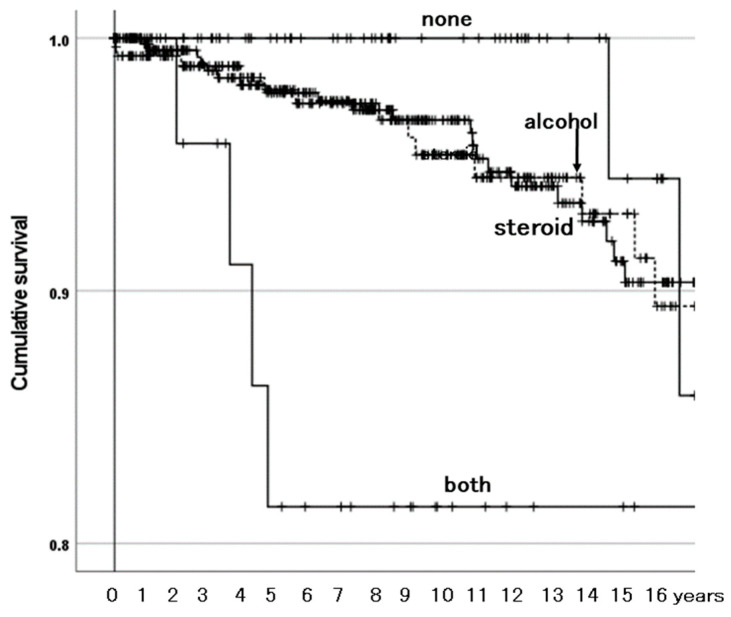
The cumulative survival of bipolar hemiarthroplasties (BPs) among osteonecrosis of the femoral head (ONFH)-associated factor groups with need for reoperation as the endpoint. Steroid: systemic steroid use. Alcohol: excessive alcohol consumption. Both: both ONFH-associated factors. None: no ONFH-associated factors. Survival rates with none, steroid, alcohol, and both were 100%, 97%, 95%, and 82% at 10 years and 94%, 90%, 93%, and 82% at 15 years, respectively. The group with both ONFH-associated factors had lower survivorship than the other groups (*p* ≤ 0.001). The other groups were not different from one another in survivorship (*p* ≥ 0.075).

**Figure 4:**
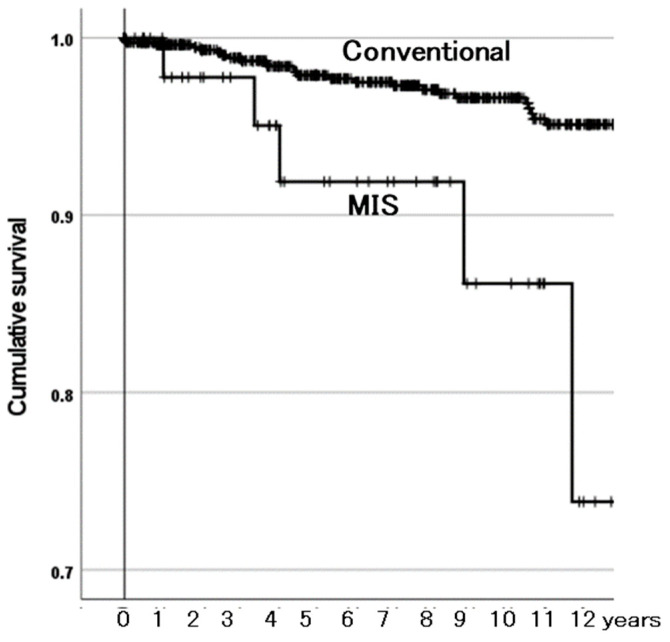
The cumulative survival of bipolar hemiarthroplasties (BPs) between incision length groups with need for reoperation as the endpoint. Conventional: conventional incision. MIS: minimum-incision surgery. Their survival rates were 97% and 86% at 10 years, respectively. The MIS group had lower survivorship than the conventional group (*p* < 0.001).

**Figure 5:**
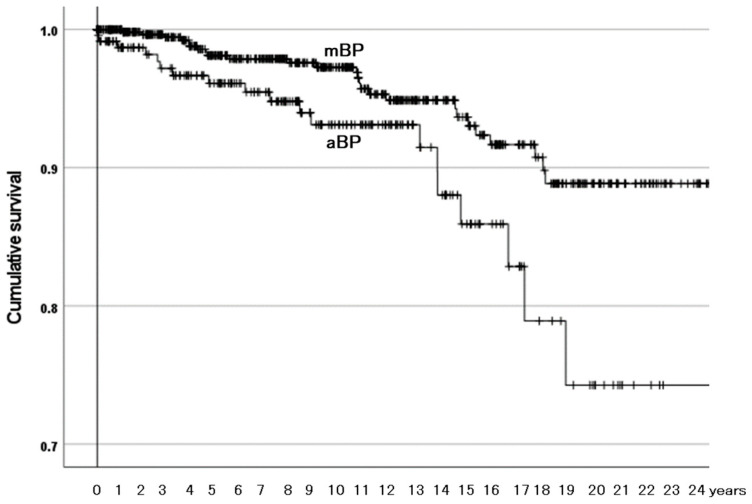
The cumulative survival of bipolar hemiarthroplasties (BPs) between the groups of materials of the outer surface of the outer head with need for reoperation as the endpoint. mBP: BPs with a metal outer head. aBP: BPs with an alumina outer head. Their survival rates were 97% and 93% at 10 years and 89% and 74% at 20 years, respectively. The aBPs had lower survivorship than the mBPs (*p* = 0.008).

The Kaplan–Meier estimator illustrated the effects of the three identified risk factors on the survivorship of BPs with need for reoperation as the endpoint. Among the ONFH-associated factor groups, the group with both factors was at a higher risk than the other groups (Figure 3). The other groups were not different from one another in survivorship. The MIS group had lower survivorship than the conventional incision group (Figure 4). The aBPs had lower survivorship than the mBPs (Figure 5). The rates of need for reoperation were also different between them in a χ^2^-test (*p* = 0.014): 4.2% in the mBPs and 8.4% in the aBPs. In both groups, the proximal migration of the outer head was the main reason for need for reoperation in 17 of 27 (63.0%) of the mBPs and 13 of 20 (65.0%) of the aBPs (Table 7).

## 4. Discussion

In the previous report, regarding need for reoperation, analyses of 7393 arthroplasties including 6284 THAs, 886 BPs, 188 tRSs, and 35 hRSs identified the following risk factors: both ONFH-associated factors, a lateral approach, aBPs, and hRSs [13]. However, it was not clear which risk factors were applicable to which surgical treatments. In the present study, further analyses performed separately for the 6284 THAs and 886 BPs revealed that risk factors were specific to each surgical group: acetabular-articulating material in the THAs and having both ONFH-associated factors, MIS, and aBPs in the BPs.

### 4.1. Acetabular-Articulating Material Is a Determinant of Survivorship of THAs

In the Australian registry, the cumulative percent revision of primary THAs performed for OA was higher with cPE than with HXLPE, and it was higher with metal-on-metal bearing than with metal-on-HXLPE bearing, but it was not different between ceramic-on-ceramic and metal-on-HXLPE bearings [4]. In a cohort study of 253 metal-on-metal THAs with a median follow-up of 11.5 years, 34 hips were revised, with survival rates of 89.6% at 10 years and 82.9% at 14.6 years, and 19 of them (55.9%) were revised due to ARMD [14]. In the present study, compared with the HXLPE or MXLPH group, the cPE and the metal groups had inferior survivorship; survival rates with HXLPE or MXLPH, cPE, and metal were 90%, 78%, and 83% at 20 years, respectively, whereas that of the ceramic group did not differ (Figure 2). Reasons for need for reoperation in the HXLPE or MXLPE group included recurrent dislocation at the top, followed by periprosthetic femoral fracture (Table 4). In the ceramic group, recurrent dislocation was also at the top. In the cPE group, the most frequent three reasons were osteolysis, polyethylene wear and/or breakage, and recurrent dislocation, which could be related, to some extent, to the inferior properties of cPE compared with those of HXLPE or MXLPE. In the metal group, ARMD was the top reason. Therefore, acetabular-articulating material was an important determinant of the survivorship of the THAs.

### 4.2. Combined Existence of Both ONFH-Associated Factors Affects Survivorship of BPs

Systemic steroid use [6] and excessive alcohol consumption [15,16] were risk factors associated with reoperation in THAs for ONFH. In the present study, the combined existence of both factors was a risk factor related to need for reoperation not in the THAs but in the BPs. This is reported for the first time to our knowledge.

### 4.3. MIS Affects Survivorship of BPs

As for MIS, in a report based on data from the Norwegian Arthroplasty Register, the revision rates associated with the MIS anterior and anterolateral approaches were not increased compared with those of the conventional posterior and direct lateral approaches [17]. We could not find any study that analyzed influence of MIS on the reoperation risk of BPs. However, the reoperation risk of the BPs performed with MIS was higher than that of the PBs performed using conventional incisions.

### 4.4. The Material of the Outer Head Articulating with Cartilage Is a Determinant of Survivorship of BPs

The patho-mechanism of the inferior durability of the aBPs was discussed in the previous report [13]. In short, articulation with the BP outer head could be harmful to the acetabular cartilage, and the better lubrication of cartilage with aBP than with mBP could increase articulation in aBP, leading to the proximal migration of the outer head and pain, resulting in a higher risk of reoperation in aBPs than in mBPs.

### 4.5. Little Difference Based on Component Fixation in Survivorship of THAs

Component fixation has been many hip surgeons’ interest in relation to the durability of hip prostheses. In the Australian registry, in the cumulative percent revision of primary THAs performed for OA, there was little difference in outcomes based on fixation (cemented, hybrid, or cementless), except for patients aged ≥ 75 years, where the revision rate was lower when either hybrid or cemented fixation was used [4]. As for younger patients, in a study analyzing the most commonly used uncemented, hybrid, and fully cemented implant combinations in the New Zealand registry, in 40–55-year-old patients, revision rates were comparable between the uncemented and the hybrid implant combinations, whereas the cemented implant combination exhibited a higher revision rate. In <40-year-old patients, the revision rate for the hybrid implant combination was significantly lower than that for the uncemented implant combination [18]. In the present study of THAs and BPs performed for ONFH, fixation (uncemented/cement and surface finish of femoral stem) was not associated with need for reoperation.

### 4.6. Limitations

Some patient-related and operative data were lacking, e.g., surgical experience. Hip arthroplasty practice changed over the observation period in surgical approach, component fixation, acetabular-articulating material, and the material and diameter of the femoral head, although all of them were analyzed in the present study. Information on complications unrelated to reoperation could not be obtained, given the constraints involving the 31 institutions. THAs and BPs performed only for Japanese ONFH patients were analyzed. All of them warrant further research.

## 5. Conclusions

The present further analyses performed separately for the 6284 THAs and 886 BPs revealed that risk factors associated with need for reoperation were specific to each surgical group: acetabular-articulating material in the THAs and both ONFH-associated factors, MIS, and aBPs in the BPs. The articulating materials, i.e., the acetabular-articulating material in the THAs and the alumina outer surface of the outer head in the BPs, were important determinants in the survivorship of hip prostheses in ONFH patients.

## Figures and Tables

**Table 1:** Comparison of characteristics (means ± SDs or percentages) of 6284 THAs and 886 BPs performed for ONFH using *t*-test and χ^2^ test.

Variable		THAs	BPs	*p*-Value
Patient age (years)		52.0 ± 14.34	49.2 ± 14.21	<0.001
Male gender (%)		54.0	58.1	0.024
Height (cm)		162.1 ± 9.26	163.0 ± 9.10	0.011
Weight (kg)		61.3 ± 12.84	60.8 ± 11.83	0.242
BMI (body mass index) (kg/m^2^)		23.23 ± 3.951	22.80 ± 3.589	0.002
ONFH-associated factor (%)	Systemic steroid use	59.9	53.9	<0.001
	Excessive alcohol consumption	27.0	33.5	
	None of them	10.8	9.3	
	Both of them	2.3	3.3	
ONFH stage (%)	2 (without collapse of FH)	2.7	4.6	<0.001
	3 (collapse of FH)	49.8	89.6	
	4 (osteoarthrosis)	47.5	5.8	
Previous surgery in index joint (%)	Yes (joint-preserving surgery)	8.1	4.3	<0.001
Surgical approach (%)	Posterior	58.5	87.6	<0.001
	Anterior or anterolateral	22.3	1.9	
	Lateral	19.2	10.5	
Incision length (%)	Conventional	68.9	94.0	<0.001
	MIS	31.1	6.0	
Cemented fixation (%)	Acetabular component	2.3	NA	NA
	Femoral component	14.4	8.2	<0.001
Surface finish of femoral stem	Grid blast (for bone on growth)	7.8	4.3	<0.001
	Porous with/without coating	69.9	83.6	
	Cement with/without polish	13.9	7.1	
	Others	8.4	5.0	
Acetabular-articulating material in THAs and inner articulating surface of outer head in BPs (%)	HXLPE	61.4	13.1	NA
	MXLPE	21.3	0	
	cPE	9.3	86.9	
	Cobalt–chrome	4.3	0	
	Ceramic	3.7	0	
Femoral head material in THAs and inner head material in BPs (%)	Ceramic	64.4	40.2	NA
	Cobalt–chrome	28.2	53.4	
	Oxidized zirconium	5.6	1.8	
	Stainless steel	1.8	4.6	
Head diameter in THAs and inner head diameter in BPs (%)	≥36 mm	21.4	0	NA
	32 mm	36.3	0.6	
	28 mm	23.9	18.2	
	26 mm	14.5	32.5	
	22 mm	3.9	48.7	

SDs: standard deviations. THAs: total hip arthroplasties. BPs: bipolar hemiarthroplasties. ONFH: osteonecrosis of the femoral head. FH: the femoral head. MIS: minimum-incision surgery (defined as the use of a ≤ 10 cm incision to complete a hip arthroplasty). NA: not applicable. HXLPE: highly (approximately 10 Mrad) cross-linked polyethylene. MXLPE: moderately (5 to 7.5 Mrad) cross-linked polyethylene. cPE: conventional polyethylene.

**Table 2:** Univariate analysis of each variable applying the COX proportional-hazard model to the 6284 THAs performed for osteonecrosis of the femoral head (ONFH).

Variable	Factor	Hazard Ratio (95% CI)	*p*-Value
Patient age (years)	Increment of 1	1.01 (0.99–1.02)	0.146
Gender	Female	Reference	
	Male	1.02 (0.75–1.37)	0.920
Height (cm)	Increment of 1	1.00 (0.98–1.02)	0.997
Weight (kg)	Increment of 1	0.99 (0.98–1.01)	0.333
BMI (body mass index) (kg/m^2^)	Increment of 1	0.98 (0.94–1.02)	0.247
ONFH-associated factor	Systemic steroid use	0.94 (0.57–1.55)	0.807
	Excessive alcohol consumption	1.31 (0.76–2.25)	0.326
	None of them	Reference	
	Both of them	1.82 (0.72–4.60)	0.203
ONFH stage	2 or 3 (before osteoarthrosis)	0.84 (0.61–1.14)	0.253
	4 (osteoarthrosis)	Reference	
Previous hip surgery in index joint	No	Reference	
	Yes	1.16 (0.72–1.86)	0.554
Surgical approach	Posterior	0.71 (0.50–1.01	0.054
	Anterior or anterolateral	0.42 (0.23–0.79)	0.007
	Lateral	Reference	
Incision length	Conventional	Reference	
	MIS	0.53 (0.33–0.83)	0.005
Acetabular component fixation	Uncemented	Reference	
	Cemented	1.71 (0.84–3.49)	0.137
Femoral component fixation	Uncemented	Reference	
	Cemented	1.04 (0.67–1.62)	0.860
Surface finish of femoral stem	Grid blast (for bone on growth)	Reference	
	Porous with/without coating	0.81 (0.048–1.36)	0.429
	Cement with/without polish	0.73 (0.38–1.42)	0.353
	Others	0.59 (0.29–1.24)	0.164
Acetabular-articulating material	HXLPE or MXLPE	Reference	
	cPE	2.82 (2.00–3.98)	<0.001
	Metal (metal-on-metal THA)	2.12 (1.25–3.59)	0.005
	Ceramic (ceramic-on-ceramic THA)	1.11 (0.57–2.15)	0.765
Femoral head material	Metal	Reference	
	Ceramic	0.50 (0.37–0.68)	<0.001
Head diameter	≥36 mm	0.38 (0.21–0.67)	<0.001
	32 mm	0.30 (0.17–0.51)	<0.001
	28 mm	0.36 (0.22–0.58)	<0.001
	26 mm	0.41 (0.26–0.66)	<0.001
	22 mm	Reference	

THAs: total hip arthroplasties. ONFH: osteonecrosis of the femoral head. CI: confidence interval. MIS: minimum-incision surgery. HXLPE: highly (approximately 10 Mrad) cross-linked polyethylene. MXLPE: moderately (5 to 7.5 Mrad) cross-linked polyethylene. cPE: conventional polyethylene.

**Table 3:** Multivariate analysis applying the COX proportional-hazard model to the 6284 THAs performed for ONFH.

Variable	Factor	Hazard Ratio (95% CI)	*p*-Value
Surgical approach	Posterior	0.69 (0.47–1.00)	0.050
	Anterior or anterolateral	0.52 (0.27–1.00)	0.051
	Lateral	Reference	
Acetabular-articulating material	HXLPE or MXLPE	Reference	
	cPE	2.55 (1.69–3.84)	<0.001
	Metal (metal-on-metal THA)	2.15 (1.08–4.28)	0.028
	Ceramic (ceramic-on-ceramic THA)	1.27 (0.63–2.57)	0.503
Head diameter	≥36 mm	0.62 (0.28–1.36)	0.233
	32 mm	0.68 (0.35–1.32)	0.250
	28 mm	0.60 (0.34–1.03)	0.065
	26 mm	0.68 (0.40–1.13)	0.138
	22 mm	Reference	

THAs: total hip arthroplasties. ONFH: osteonecrosis of the femoral head. CI: confidence interval. HXLPE: highly (approximately 10 Mrad) cross-linked polyethylene. MXLPE: moderately (5 to 7.5 Mrad) cross-linked polyethylene. cPE: conventional polyethylene.

**Table 4:** Reasons for need for reoperation regarding acetabular-articulating materials in the 6284 THAs performed for ONFH.

Reasons for Need for Reoperation (n)	HXLPE or MXLPE (5194)	cPE (584)	Metal (274)	Ceramic (232)	Total (6284)
Recurrent dislocation	35	13	3	6	57
Periprosthetic femoral fracture	20	5	0	1	26
Osteolysis	6	18	2	0	26
Polyethylene wear and/or breakage	3	17	1	0	21
Stem loosening	9	8	0	1	18
Socket loosening	9	2	2	1	14
Adverse reaction to metal debris	1	0	8	0	9
Others (n ≤ 3 for each)	10	5	2	1	18
Total (%)	93 (1.8%)	68 (11.6%)	18 (6.6%)	10 (4.3%)	189 (3.0%)

THAs: total hip arthroplasties. ONFH: osteonecrosis of the femoral head. HXLPE: highly (approximately 10 Mrad) cross-linked polyethylene. MXLPE: moderately (5 to 7.5 Mrad) cross-linked polyethylene. cPE: conventional polyethylene.

**Table 5:** Univariate analysis of each variable applying the COX proportional-hazard model to the 886 BPs performed for ONFH.

Variable	Factor	Hazard Ratio (95% CI)	*p*-Value
Patient age (years)	Increment of 1	0.99 (0.97–1.01)	0.304
Gender	Female	Reference	
	Male	1.05 (0.59–1.88)	0.868
Height (cm)	Increment of 1	1.01 (0.98–1.05)	0.452
Weight (kg)	Increment of 1	1.01 (0.99–1.04)	0.351
BMI (kg/m^2^)	Increment of 1	1.03 (0.95–1.13)	0.480
ONFH-associated factor	Systemic steroid use	1.96 (0.46–8.28)	0.363
	Excessive alcohol consumption	2.16 (0.49–9.42)	0.308
	None of them	Reference	
	Both of them	10.34 (2.00–53.57)	0.005
ONFH stage	2 or 3 (before osteoarthrosis)	0.51 (0.20–1.30)	0.161
	4 (osteoarthrosis)	Reference	
Previous hip surgery in index joint	No	Reference	
	Yes	0.98 (0.30–3.18)	0.976
Surgical approach	Posterior	0.77 (0.32–1.81	0.546
	Anterior or anterolateral	UC1	0.974
	Lateral	Reference	
Incision length	Conventional	Reference	
	MIS	5.07 (2.07–12.39)	<0.001
New type of BPs (nBPs) or others (oBPs)	nBPs	Reference	
	oBPs	0.83 (0.46–1.51)	0.551
Surface of prosthetic neck: smooth (sBPs) or rough (rBPs)	sBPs	Reference	
	rBPs	1.40 (0.75–2.60)	0.292
Outer surface of outer head: alumina ceramic (aBPs) or metal (mBPs)	aBPs	2.17 (1.20–3.92)	0.010
	mBPs	Reference	
Inner articulating surface of outer head: HXLPE (hBPs) or cPE (cBPs)	hBPs	Reference	
	cBPs	0.56 (0.24–1.35)	0.200
Femoral component fixation	Uncemented	Reference	
	Cemented	0.94 (0.29–3.03)	0.911
Surface finish of femoral stem	Grid blast (for bone on growth)	Reference	
	Porous with/without coating	0.57 (0.14–2.37)	0.435
	Cement with/without polish	0.66 (0.11–3.98)	0.653
	Others	0.16 (0.01–1.78)	0.135
Material of inner head	Metal	Reference	
	Ceramic	1.63 (0.91–2.92)	0.101
Inner head diameter	32 mm	UC2	0.975
	28 mm	0.80 (0.33–1.96)	0.630
	26 mm	1.20 (0.62–2.31)	0.597
	22 mm	Reference	

BPs: bipolar hemiarthroplasties. ONFH: osteonecrosis of the femoral head. CI: confidence interval. BMI: body mass index. UC1: unable to calculate because no case needed reoperation in the 17 BPs performed with an anterior or anterolateral approach. MIS: minimum-incision surgery. HXLPE: highly (approximately 10 Mrad) cross-linked polyethylene. cPE: conventional polyethylene. UC2: unable to calculate because no case needed reoperation in the 5 BPs with a 32 mm inner head.

**Table 6:** Multivariate analysis applying the COX proportional-hazard model to the 886 BPs performed for ONFH.

Variable	Factor	Hazard Ratio (95% CI)	*p*-Value
ONFH-associated factor	Systemic steroid use	1.98 (0.47–8.40)	0.354
	Excessive alcohol consumption	2.14 (0.49–9.39)	0.311
	None of them	Reference	
	Both of them	9.13 (1.76–47.33)	0.008
Incision length	Conventional	Reference	
	MIS	5.61 (2.25–13.97)	<0.001
Outer surface of outer head	Alumina ceramic (aBPs)	2.40 (1.32–4.38)	0.004
	Metal (mBPs)	Reference	

BPs: bipolar hemiarthroplasties. ONFH: osteonecrosis of the femoral head. CI: confidence interval. MIS: minimum-incision surgery. aBPs: BPs with an alumina outer head. mBPs: BPs with a metal outer head.

**Table 7:** Reasons for need for reoperation regarding the materials of the outer surface of the outer head in the 886 BPs performed for ONFH.

Reasons for Need for Reoperation (n)	mBP (648)	aBP (238)	Total (886)
Proximal migration of the outer head	17	13	30
Pain	5	2	7
Others (n ≤ 3 for each)	5	5	10
Total (%)	27 (4.2%)	20 (8.4%)	47 (5.3%)

BPs: bipolar hemiarthroplasty. ONFH: osteonecrosis of the femoral head. mBP: BP with a metal outer head. aBP: BP with an alumina outer head.

## Data Availability

The data that support the findings for this study are available to other researchers from the corresponding author upon reasonable request. The data are not publicly available due to the conditions included in the ethical approvals for this study.
